# Analysis of the distribution of macronutrients of food baskets delivered by municipalities during the COVID-19 pandemic in Peru

**DOI:** 10.17843/rpmesp.2022.391.9742

**Published:** 2022-03-28

**Authors:** Bladimir Morales-Cahuancama, Gandy Dolores-Maldonado, Paul Hinojosa-Mamani, William Bautista-Olortegui, Cinthia Quispe-Gala, Lucio Huamán-Espino, Juan Pablo Aparco

**Affiliations:** 1 Centro Nacional de Alimentación y Nutrición, Instituto Nacional de Salud, Lima, Peru. Centro Nacional de Alimentación y Nutrición Instituto Nacional de Salud Lima Peru; 2 Universidad Privada de Ciencias Aplicadas, Lima, Peru. Universidad Peruana de Ciencias Aplicadas Universidad Privada de Ciencias Aplicadas Lima Peru; 3 Escuela Profesional de Nutrición, Facultad de Medicina, Universidad Nacional Mayor de San Marcos, Lima, Peru. Universidad Nacional Mayor de San Marcos Escuela Profesional de Nutrición Facultad de Medicina Universidad Nacional Mayor de San Marcos Lima Peru; 4 Oficina General de Investigación y Transferencia Tecnológica, Instituto Nacional de Salud, Lima, Peru. Oficina General de Investigación y Transferencia Tecnológica Instituto Nacional de Salud Lima Peru

**Keywords:** Food Basket, Covid-19, Emergency Feeding, Disaster Vulnerability, Food Security, Perú

## Abstract

**Objectives.:**

To evaluate and compare the macronutrient distribution of the food baskets delivered by Peruvian municipalities during the COVID-19 pandemic according to the geographic domain and assigned budget level.

**Materials and methods.:**

Secondary analysis of the database “Consultation of Acquisition and distribution of basic necessities of the basic family basket” of the General Comptroller of the Republic. Stratified probability sampling was carried out. The caloric intake distribution was calculated according to macronutrients and compared with the Acceptable Intervals of Macronutrient Distribution (IADM) of the Nutrition Institute of Central America and Panama (INCAP) and the National Institute of Civil Defense (INDECI).

**Results.:**

At the national level, the median caloric intake of proteins was 7.7%; for carbohydrates it was 62.5%; and for fats it was 28.1%. The proportion of municipalities with protein deficit was 84%; Municipalities with excess carbohydrates ranged from 16.5% (according to INCAP) to 35.9% (according to INDECI), and with excess fat, it was between 61.6% (according to INCAP) and 20.2% (according to INDECI). According to INDECI, nationally only 9.2% of municipalities delivered baskets with an adequate distribution of macronutrients; Metropolitan Lima stands out with the highest proportions of adequate baskets, while in the Jungle region this percentage was less than 5%.

**Conclusions.:**

Most of the baskets delivered did not have adequate macronutrient distribution. Especially the baskets of municipalities outside of Metropolitan Lima or those that had smaller budget. Carbohydrates and fats were the nutrients that were included in excess, while proteins were deficient.

## INTRODUCTION

The COVID-19 pandemic has significantly affected health systems and the quality of life of the world’s population ^1^. On March 16, 2020, the Peruvian government declared a nationwide state of emergency and implemented social isolation ^2^, which limited economic activities and exacerbated the social vulnerability of many households ^3^. In addition, the pandemic affected access to food and markets became focal points of infection, leading to the closure or the reduction of capacity. Likewise, the decrease of family income and price speculation affected access to food ^4^. These changes worsened food insecurity (FI) in Peru, which was already a complex problem before the pandemic ^5^; thus, for the period 2014-2016 the prevalence of FI was estimated at 50.7% and for 2018-2020 it increased to 67% ^6^, although other authors have estimated up to 83% FI in 2020 ^7^.

In the face of this scenario of economic crisis and FI due to the pandemic, the central government transferred 213 million soles, equivalent to US$59.6 million (exchange rate as of July 2020) to the municipalities for the purchase of food for the basic family food basket, in order to attend, on a one-time basis, to households in vulnerable situations ^8^. The money distribution was proportional to the number of inhabitants of each municipality. In addition, the Guidelines for the Organization and Distribution of Food Baskets (LODCA) were published to guide municipalities on the details of the program ^9^ and currently recommends the inclusion of food according to the geographical area and consumption habits of the population.

Food assistance in response to humanitarian crises has proven to be beneficial for affected populations ^10^ ^,^ ^11^. Entities such as the Institute of Nutrition of Central America and Panama (INCAP) ^12^ and the National Institute of Civil Defense (INDECI) ^13^ recommend the composition of emergency food baskets based on food and nutrition criteria, using acceptable macronutrient distribution ranges (AMDRs) that express recommendations for protein, carbohydrates and fats as a percentage of total caloric intake. The AMDRs are estimated to ensure sufficient nutrient intake without increasing the risks of chronic diseases, and allow understanding whether the food assistance is nutritionally balanced, which is the purpose of the analysis ^14^.

Although the COVID-19 pandemic continues and several interventions are still needed, including the distribution of emergency food baskets, to date there has been no evaluation of the nutritional composition or the adequacy of the food baskets delivered during the health emergency. Therefore, this study aimed to evaluate and compare the distribution of macronutrients in the food baskets delivered by municipalities during the COVID-19 pandemic according to geographic domain and level of allocated budget.

**KEY MESSAGES**

Motivation for the study: Nutritional criteria should be applied during the preparation of food baskets distributed to the population with limited food access.

Main findings: Most food baskets provided by municipalities were not nutritionally balanced. Carbohydrates and fats were the nutrients included in excess, while proteins were in deficit.

Implications: It is necessary to improve the guidelines for the preparation of emergency food baskets considering the acceptable ranges of macronutrient distribution and to ensure adequate food baskets, as well as to seek strategies to increase the amount of food sources of protein and to regulate the amount of fats in the emergency baskets.

## MATERIALS AND METHODS

### Study design

This article is a secondary analysis of the database “Acquisition and distribution of basic necessities of the basic family basket” of the Office of the Comptroller General of the Republic of Peru (CGRP) ^15^.

### Population, sample and sampling

The study population consisted of the 1874 municipalities registered in the CGRP web page. The sample size was calculated based on an expected proportion of 50% (maximum variance), resulting in a net sample of 319 municipalities; in addition, 30% was added for loss (municipalities with errors in the registration of baskets).

The municipalities were selected by stratified probability sampling according to geographic area: Metropolitan Lima (differentiated from the coast), rest of the coast, highlands, and jungle. They were also classified according to the amount of allocated money ^9^: Group A (50,000 soles or US$14,000); Group B (100,000 soles or US$28,000); Group C (200,000 soles or US$56,000) and Group D (more than 500,000 soles or US$140,000).

**Figure 1:**
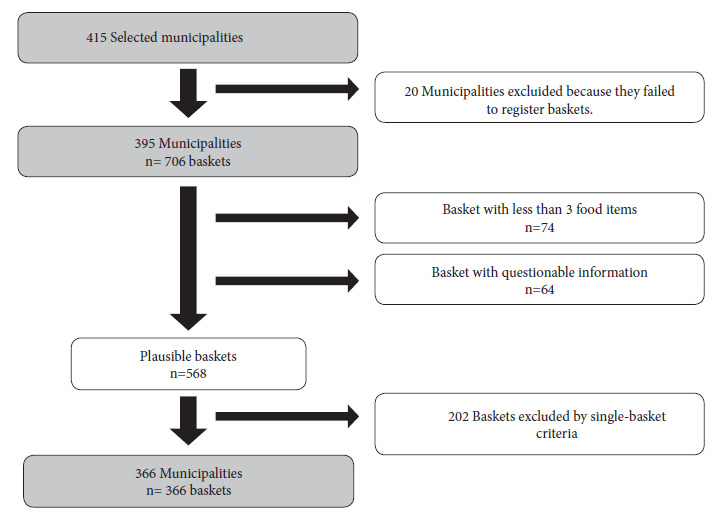
Flow chart for the collection of information from the food baskets of the selected municipalities

We included only one food basket from each selected municipality that met the inclusion criterion food baskets made up of at least three foods; in the event that the municipality had more than one type of food basket, we chose the one that was distributed to the largest number of beneficiaries. Food baskets with incorrect records were excluded, such as those that included excessive quantities of food and information with extreme values of weights or volumes of food. Fifty-three percent of the selected municipalities had more than one type of basket (Figure 1).

### Procedures

The extraction of the information from the web (from June 17 to June 21, 2020) was conducted after the deadline that the municipalities had to register the information (May 30, 2020). Subsequently, four nutritionists analyzed the quality of the collected information in order to verify food weights and that the data were complete. With the available information, we prepared a database that contained the name and weight or volume of the food.

In the case of foods that were registered on the web as “units” (without reporting the weight), we verified the price of each unit and the weight was approximated by considering the average cost of the product in wholesale and retail markets ^16^. Most of the weight approximations were made for meats (52.2%), because several canned fish were not registered with the net weight of the product but in “units”. Codes were then assigned to each food according to the Peruvian Tables of Food Composition (TPCA)^ (^ ^17^; some foods had no code in the TPCA, and were assigned the code of a similar food (similarity approximation). Most of the similarity approximations were also made for meats (67.5%), since many varieties of canned fish do not have a specific code in the TPCA.

After obtaining the TPCA codes and quantities (grams or liters) of all foods, we calculated the amount of energy (kcal), protein (g), fat (g) and carbohydrate (g) contained in each food. Subsequently, the amount of energy and the three macronutrients (g) of each basket were calculated by adding the contributions of all foods without excluding any.

Likewise, foods were classified according to groups recommended by the “Food Guide for the Peruvian Population” ^18^: sugars, meats, cereals, oils, dairy products, legumes, fruits, eggs, vegetables and tubers.

### Variables and measurements

The distribution of caloric intake according to macronutrients was calculated as the quotient of the energy intake of the macronutrients in the basket, multiplied by 100 to obtain the percentage; in addition, to estimate the available energy, we applied the Atwater factors for proteins, fats and carbohydrates: 4, 9 and 4 kcal respectively ^19^. 

To evaluate whether the baskets had a balanced dietary composition, the distribution of the caloric intake of macronutrients was compared according to the international AMDRs of INCAP, which recommends a protein content of 10-15% of the total energy content (kilocalories), 20-25% for fats, and 60-70% for carbohydrates ^12^; and the national AMDRs of INDECI, which suggest a protein intake of 10-15% of the total energy content, 20-35% for fats and 50-65% for carbohydrates ^13^.

### Statistical analysis

We calculated absolute and relative frequencies for categorical variables, while results from quantitative variables were expressed as median and interquartile range. In addition, the Kolmogorov-Smirnov test was applied to the variables “caloric intake of macronutrients” and “number of food groups”, which did not have a normal distribution. The Kruskal-Wallis test was used to compare caloric intake and the number of food groups according to geographic location and type of budget allocated, and the Dunnett’s test was used for* post hoc* comparisons between groups. Statistical significance was taken as p<0.05. We used SPSS 25.0.1 software for the statistical analysis.

### Ethical Aspects

This study was carried out using a secondary public database. Information was obtained from the “Official report on the purchase and delivery of the basic family basket” of the CGRP web page available at https://emergenciasanitaria.contraloria.gob.pe/ ^15^.

## RESULTS

The study began with 415 municipalities and after applying the exclusion criteria we obtained 366 municipalities with their respective food baskets, which allowed us to exceed the net sample size (Figure 1).

The median caloric intake provided by proteins was 7.7%, for carbohydrates it was 62.5% and for fats it was 28.1% of total calories. According to the geographic area, the highlands and the jungle had the lowest caloric intake of protein, with significant differences when compared to Metropolitan Lima and the rest of the coast (p<0.001). Metropolitan Lima had the lowest caloric intake of carbohydrates (p<0.05). The rest of the coast had the lowest caloric intake of fats compared to the rest of Peru (p<0.05). On the other hand, the baskets of municipalities with the largest budget (Group D) had significantly higher caloric intake from proteins and lower intake from carbohydrates (p<0.05); while the baskets of municipalities in Group B showed significantly higher caloric intake from fats (Table 1).

**Table 1:** Percentage of caloric intake of food baskets distributed by municipalities according to geographic domain and budget.

Municipalities	Caloric intake by macronutrients		
	Proteins	Carbohydrates	Fats
	Me (IQR)	Me (IQR)	Me (IQR)
Geographical domain			
Rest of the coast	8.5 (7.4 - 9.6) ^a^	62.3 (58.6 - 67.0) ^a^	26.3 (21.2 - 31.4) ^a^
Highlands	7.4 (5.8 - 8.8) ^b^	62.9 (56.9 - 67.9) ^a^	28.5 (21.4 - 33.8) ^b^
Jungle	7.4 (6.0 - 8.8) ^b^	62.3 (57.8 - 67.7) ^a^	28.1 (23.1 - 34.0) ^b^
Metropolitan Lima	10.7 (9.3 - 11.3) ^c^	57.8 (54.4 - 61.5) ^b^	29.4 (24.7 - 30.6) ^ab^
Budget			
Group A (14,000 USD)	7.7 (6.1 - 8.9) ^a^	63.8 (57.8 - 68.4) ^a^	27.3 (21.5 - 33.2) ^a^
Group B (28,000 USD)	7.4 (5.9 - 9.0) ^a^	61.6 (55.1 - 67.1) ^b^	30.1 (22.2 - 35.2) ^b^
Group C (56,000 USD)	7.6 (6.4 - 9.5) ^a^	62.8 (57.9 - 66.8) ^ab^	27.3 (23.3 - 31.6) ^a^
Group D (>140,000 USD)	10.7 (9.0 - 11.6) ^b^	58.8 (54.1 - 61.5) ^c^	25.0 (24.2 - 29.4) ^a^
National	7.7 (6.2 - 9.2)	62.5 (57.1 - 67.4)	28.1 (21.8 - 33.7)

Kruskal-Wallis, Dunnett’s *post-hoc* test. Values with different letters represent significant differences between comparison groups, p<0.05

Me: Median, IQR: Interquartile range.

Table 2 shows the analyzed quantities according to food groups. Meats were the group with less presence in all the food baskets (0.7 kg), while cereals were the most abundant (13.1 kg). The municipalities of Metropolitan Lima provided the least amount of sugar in their food baskets, while municipalities in the highlands included the most sugar, 1.5 kg (p<0.05). The rest of the coast and the jungle included the least amount of meat compared to the other domains, 0.7 kg (p<0.05). The highlands and the jungle included the most oil (p<0.05). The highlands included the largest amount of legumes in their baskets, 3 kg. The baskets of the municipalities with the lowest budgets (Group A) had a greater amount of sugar, cereals, oils, dairy products and legumes, with significant differences with the municipalities with the highest budgets (p<0.05).

**Table 2:** Quantity by food group of the baskets according to geographic domain and budget.

Municipalities	Sugar (kg)	Meats* (kg)	Cereal (kg)	Oils (L)	Dairies** (kg)	Legumes (kg)
	Me (IQR)	Me (IQR)	Me (IQR)	Me (IQR)	Me (IQR)	Me (IQR)
Geographical domain						
Rest of the coast	3.0 (2.0 - 5.0) ^a^	0.7 (0.5 - 0.9) ^a^	11.5 (9.3 - 13.4) ^a^	1.0 (1.0 - 2.0) ^a^	1.6 (1.2 - 2.0) ^a^	2.0 (1.0 - 2.5) ^a^
Highlands	5.0 (3.8 - 7.0) ^b^	0.8 (0.5 - 1.2) ^b^	14.3 (11.0 - 19.0) ^b^	2.0 (1.5 - 3.0) ^b^	1.6 (1.2 - 2.4) ^a^	3.0 (2.0 - 4.0) ^b^
Jungle	3.0 (3.0 - 5.0) ^a^	0.7 (0.5 - 0.9) ^a^	13.0 (10.9 - 16.0) ^c^	2.0 (1.0 - 2.0) ^c^	1.2 (1.0 - 2.0) ^ab^	2.0 (1.0 - 3.0) ^a^
Metropolitan Lima	1.5 (1.0 - 2.0) ^c^	1.0 (1.0 - 1.2) ^c^	10.0 (7.5 - 10.4) ^d^	1.0 (1.0 - 2.0) ^a^	1.6 (1.2 - 2.0) ^a^	2.0 (2.0 - 2.3) ^a^
Budget						
Group A (14,000 USD)	5.0 (4.0 - 8.0) ^a^	0.8 (0.5 - 1.2) ^a^	14.5 (11.0 - 20.0) ^a^	2.0 (1.5 - 3.0) ^a^	1.6 (1.2 - 2.4) ^a^	2.6 (2.0 - 4.0) ^a^
Group B (28,000 USD)	4.0 (3.0 - 5.7) ^b^	0.8 (0.5 - 1.0) ^ab^	13.4 (11.0 - 16.0) ^b^	2.0 (1.0 - 3.0) ^a^	1.6 (1.2 - 2.0) ^a^	2.0 (2.0 - 3.3) ^a^
Group C (56,000 USD)	3.0 (2.0 - 5.0) ^c^	0.7 (0.5 - 1.0) ^c^	12.0 (9.0 - 14.0) ^c^	1.8 (1.0 - 2.0) ^b^	1.2 (0.8 - 1.6) ^b^	2.0 (1.8 - 3.0) ^b^
Group D (>140,000 USD)	1.5 (1.0 - 2.0) ^d^	0.9 (0.7 - 1.0) ^a^	9.5 (8.0 - 10.0) ^d^	1.0 (1.0 - 1.0) ^c^	1.6 (1.2 - 2.0) ^ab^	2.0 (2.0 - 4.0) ^ab^
National	5.0 (3.0 - 6.0)	0.7 (0.5 - 1.0)	13.1 (10.0 - 17.0)	2.0 (1.0 - 3.0)	1.6 (1.2 - 2.0)	2.0 (2.0 - 4.0)

Kruskal-Wallis, Dunnet *post-hoc* test. Values with different letters represent significant differences between comparison groups, p<0.05.

Me: Median. IQR: Interquartile range.

*The meat group consisted mostly of canned fish preserves.

**The dairies group consisted mostly of canned evaporated milk.

For presentation purposes only, the columns referring to the food groups: fruits, eggs, vegetables and tubers were not included in Table 2 because very few baskets included these foods and in many categories the medians were "0". All foods were included in the calculation of caloric and macronutrient intake.

According to the AMDRs of INCAP and INDECI, the proportion of municipalities with protein deficit was 84%, while the proportions of those with deficit of carbohydrates and fats were 39.8% and 18.1% according to INCAP and 7.5% and 18.1% according to INDECI. No excess of protein was found, but the excess of carbohydrates ranged from 16.5 to 35.9%, according to the applied criteria. Similarly, the excess of fats ranged from 20.2 to 61.6% (Table 3).

**Table 3:** Adaptation of the caloric intake of macronutrients to INCAP and INDECI standards according to geographic area and budget.

Municipalities	INCAP / INDECI	INCAP		INDECI		INCAP		INDECI	
	Protein deficit n (%)	Carbohydrate deficit n (%)	Excess carbohydrate n (%)	Carbohydrate deficit n (%)	Excess carbohydrate n (%)	Fat deficit n (%)	Excess fat n (%)	Fat deficit n (%)	Excess fat n (%)
Geographical domain									
Rest of the coast	228 (82)	93 (33.6)	39(13.9)	19 (6.8)	95 (34)	57 (20.7)	155 (55.7)	57 (20.7)	43 (15.3)
Highlands	1067 (86)	498 (40.2)	247 (19.9)	113 (9.1)	471 (38)	248 (20)	780 (62.9)	248 (20)	254 (20.5)
Jungle	266 (86.9)	121 (39.4)	24 (7.9)	6 (1.9)	102 (33.3)	35 (11.4)	189 (61.9)	35 (11.4)	73 (23.8)
Metropolitan Lima	15 (29.8)	35 (70.4)	0 (0)	4 (8.8)	6 (12)	0 (0)	31 (61.6)	0 (0)	9 (18.8)
Budget									
Group A (14,000 USD)	790 (86.9)	330 (36.3)	179 (19.7)	63 (6.9)	375 (41.2)	180 (19.8)	547 (60.1)	180 (19.8)	176 (19.4)
Group B (28,000 USD)	419 (86.5)	219 (45.2)	71 (14.7)	58 (12)	156 (32.3)	96 (19.8)	320 (66.1)	96 (19.8)	127 (26.2)
Group C (56,000 USD)	350 (80.7)	167 (38.5)	55 (12.6)	16 (3.7)	137 (31.6)	60 (13.7)	265 (61.1)	60 (13.7)	65 (15)
Group D (>140,000 USD)	17 (36)	32 (67.4)	6 (11.7)	4 (9.4)	6 (11.7)	6 (11.7)	23 (48.5)	6 (11.7)	11 (24.3)
National	1576 (84)	748 (39.8)	310 (16.5)	142 (7.5)	674 (35.9)	340 (18.1)	1155 (61.6)	340 (18.1)	379 (20.2)

Summary of AMDRs according to INCAP and INDECI:

Protein deficit INCAP and INDECI (<10%)

Carbohydrate deficit INCAP (<60%); Excess carbohydrate INCAP (>70%)

Carbohydrate deficit INDECI (<50%); Excess carbohydrate INDECI (>65%)

Fat deficit INCAP (<20%); Excess fat INCAP (>25%)

Fat deficit INDECI (<20%); Excess fat INDECI (>35%)

Regarding geographic location, in Metropolitan Lima the protein deficit was 29.8%, while in the other areas it was greater than 80%. Metropolitan Lima had a 70.4% deficit of carbohydrates and no basket had excess (according to INCAP). The municipalities of Metropolitan Lima did not have a deficit of fats, and excessive fat intake was observed in all geographical areas. We also found that municipalities with higher budget allocations (Group D) had fewer problems of protein deficit, excess carbohydrates and excess fats, compared to municipalities with lower budgets (Table 3).

**Figure 2:**
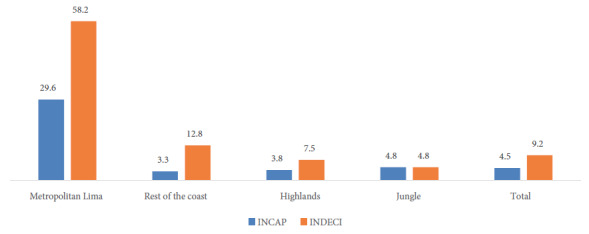
Percentage of food baskets with adequate distribution of carbohydrates, proteins and fats, by geographic domain.

Figure 2 shows that, according to INDECI criteria, only 9.2% of municipalities nationwide provided baskets with adequate distribution of the three macronutrients; Metropolitan Lima stood out with the highest proportion of adequate baskets, while this percentage was less than 5% in the jungle. After applying the INCAP parameter, we observed that less than 5% of the total baskets complied with the AMDRs, while the rest of the coast was the region with the lowest adequacy with only 3.3% of adequate baskets. 

**Figure 3:**
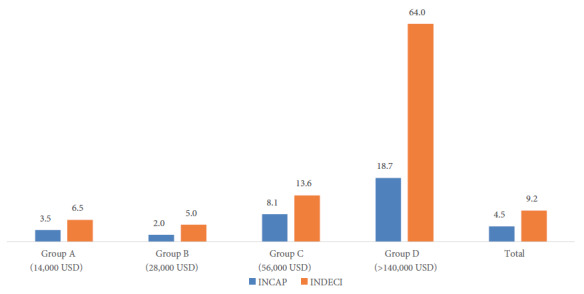
Percentage of food baskets with adequate distribution of carbohydrates, proteins and fats according to budget level.

Figure 3 presents the percentage of baskets that complied with the adequate distribution of the three macronutrients according to budget level, and it shows that the municipalities with the lowest budgets had the lowest levels of adequacy. According to INDECI criteria, 64% of municipalities in Group D distributed adequate baskets, while in groups A and B only 6.5% and 5% complied with the AMDRs; applying INCAP parameters shows that the same groups of municipalities stand out.

## DISCUSSION

This study aimed to evaluate the distribution of macronutrients such as energy, fats and proteins in the food baskets distributed by local governments to address the FI due to the COVID-19 pandemic. The findings show that less than 10% of the evaluated municipalities nationwide provided food baskets that complied with the AMDRs, according to INDECI; however, when applying the INCAP criteria, compliance dropped to less than 5%. Regarding the geographic domain, municipalities in the jungle and highlands had the lowest macronutrient adequacy. Regarding budget level, less than 7% of the baskets from municipalities in groups A and B (with the lowest budget) complied with the guidelines.

This would be the first study to analyze compliance to AMDRs in food baskets delivered during the COVID-19 pandemic in Peru. Some studies on food baskets conducted before the pandemic focused on proposing new basic food baskets (BFB); thus, a study in Costa Rica in 2013 compared the nutritional profile of the most consumed foods (current BFB) with the recommendations for daily nutrient intake to develop an optimized proposal (recommended BFB) ^20^; similarly, in Guatemala, a new BFB was constructed to adjust the nutrient intake. Other studies, also before the pandemic, analyzed the BFB from an economic perspective, reporting the variation in the price of the BFB and its causes ^21^ or the cost of a healthy BFB ^22^.

The results of our study show that most of the municipalities did not comply with an adequate distribution of macronutrients and that there are differences between the baskets distributed according to geographic area and budget level. Regarding the inadequate distribution of macronutrients, in municipalities outside Metropolitan Lima and with smaller budgets, protein was the most deficient macronutrient, with deficit proportions above 80% (89 and 86%, respectively); these same municipalities showed the highest percentage of excess carbohydrates. On the other hand, the baskets prepared by the municipalities of Metropolitan Lima and those with the largest budgets were characterized by a higher caloric contribution from proteins and a lower contribution of calories from carbohydrates.

Regarding food quantity, the baskets from municipalities in the highlands and jungle stood out for having a greater quantity of sugar, cereal and oils. Municipalities with lower budgets had similar behavior. Municipalities provided almost the same amount of meat; however, by incorporating foods such as sugar and vegetable oil, foods that do not provide other nutrients besides calories, they altered the macronutrient balance to the detriment of protein. This situation meant that the municipalities that provided more food had less macronutrient adequacy.

In addition, it is important to mention the difference in the adequacy of macronutrients in the baskets according to the budget allocated to the municipalities, considering that municipalities with larger budgets have more management personnel. The results could explain a decrease in the quality of spending the smaller the municipality. This situation could be explained by the fact that management staffs tend to be limited, as well as the local food supply.

Foods that provide more protein, such as meats, milk and eggs, were the least used. Although foods of animal origin are more expensive compared to vegetables, the cost could be reduced by opting for more economical presentations, such as canned foods in large containers, or milk in containers that are cheaper than cans. It is also necessary to develop other animal food alternatives that can be safe to transport, have lower packaging cost or change the type of meat to a more economical one.

Another important aspect is the differences in the AMDRs between the standards we used for the analysis. Both AMDRs are similar for protein recommendations; however, INCAP has a more tolerable range for carbohydrates, and INDECI has a more tolerable range for fats. Although INCAP AMDRs were developed as an instrument for defining the poverty line, they are also used to estimate food assistance requirements in emergency situations. Suggestions from people responsible for the elaboration of the BFB in different countries were taken into account during the elaboration of the AMDRs. INDECI’s AMDRs were developed in response to emergencies or disasters at the national level, with the collaboration of international institutions such as the World Food Program (WFP).

An important aspect to consider is that the current FI context is different from previous scenarios of chronic FI due to disruption of food accessibility or transitory FI due to political, social or natural events that interrupt food availability and access ^23^. The sudden onset of the COVID-19 pandemic imposed three interrelated dynamics that affected food security and caused the crisis: interruption of food supply chains at all levels, loss of income among the population, especially the poorest, and the increase in food prices due to interruptions, speculation by traders and excessive purchases out of anxiety ^24^. In this context, the distribution of food baskets would be more effective than financial support through economic vouchers, given that the dynamics of the pandemic affected not only access to food but also availability, therefore, having more money would not solve food shortages ^25^.

It is also necessary to consider that the food baskets distributed in response to the COVID-19 health emergency were not intended to cover the daily nutritional requirements of beneficiary families, unlike the emergency baskets proposed by INCAP ^12^ or INDECI ^13^, which seek to cover the requirements in temporary FI due to natural disasters and climatic events in targeted areas; these circumstances are different from the scenario of the COVID-19 pandemic that affected food systems and reduced economic income throughout the country, with long-lasting effects. However, the macronutrient distribution evaluation criteria used in this study are guidelines available from institutions with international experience in the formulation of emergency food baskets.

In a context where the risks of complications and mortality from COVID-19 are related to diseases related to overweight and obesity ^26^, it is necessary to monitor the messages and dietary interventions delivered to the population to avoid an increase in NCDs ^27^; in this sense, the delivery of baskets with excess sugar and oil could have a harmful communicational effect on the population, as well as encouraging the preparation and consumption of sweet, fried or high-fat foods.

From this perspective, it is important to design nutritionally adequate BFB to guide food consumption in the population. It is also important to design food baskets for emergencies due to climatic events or natural disasters as well as to develop other ways of delivering food under global pandemic conditions that could provoke a new food crisis, as well as COVID-19; similar likely scenarios of food security affectation by avian influenza ^28^ and Ebola ^29^ viruses have been reported.

On the other hand, the last food assistance summit emphasized the need to innovate in the development of foods that improve critical aspects of food assistance, such as low protein intake and the risk of chronic diseases resulting from the consumption of high-calorie foods ^30^. In the national context, we suggest to promote the innovation of food products that help to improve the nutritional profile of food baskets. In this regard, the macronutrient balance could be improved with legumes, since they are a good source of vegetable protein. Its low inclusion in the baskets provided by the municipalities is striking, since the national market is permanently supplied due to the high production in different areas of the country, especially in the northern and central coast of Peru ^31^. In addition, this situation could be improved by providing information to improve the preparation of baskets. In the “Guidelines for the Organization and Distribution of Food Baskets”, the information related to the nutritional composition of the basket was very limited ^9^.

The secondary study of the database has limitations due to the fact that statistical analyses are subject to the variables available and the quality of the information; in our case, the registered information on the baskets did not undergo a validation process. In addition, at the time of collecting the information from the CGRP web page, we detected that some municipalities did not register the information of their baskets. However, this did not affect the representativeness of the sampling, because we considered a loss rate during sample calculation, complying with the minimum required. Also, several municipalities inaccurately registered data about the foods in the baskets, so nutritionists were the ones who determined the approximate weight according to the unit price of the food and, in some cases, the analysis of the basket was discarded when many errors were found. One limitation in the macronutrient analysis was that the type of fats and carbohydrates could not be analyzed because the TPCA does not include this information and the nutritional labels of the foods do not provide this data. Another limitation was that the evaluation of food baskets is not a direct indicator of nutrient consumption among household members. Because the basket is not intended to cover the nutritional requirements of each individual, they were able to add other foods to their diet outside the basket; furthermore, it was not verified that families consumed the entire basket. However, this was the only information on the food response to the COVID-19 emergency and allows us to approximate the nutritional situation.

In conclusion, this study indicates that most of the food baskets distributed by the Municipalities that were not from Metropolitan Lima or those with a lower budget did not have an adequate distribution of macronutrients; carbohydrates and fats were the nutrients that were included in excess, while proteins had a deficit of more than 80%. It is necessary to review and improve the “Guidelines for the Organization and Distribution of Food Baskets in Peru” to include criteria for the preparation of emergency food baskets considering the AMDRs in order to ensure an adequate food basket; and also, to seek strategies to increase the amount of protein sources in emergency food baskets and regulate the amount of fats.
