# Supplementary material for: Analysis of the distribution of macronutrients of food baskets delivered by municipalities during the COVID-19 pandemic in Peru
Source: Rev Peru Med Exp Salud Publica. 2022 Mar 28;39(1):6–14. doi: 10.17843/rpmesp.2022.391.9742 (PMC11397761; doi:10.17843/rpmesp.2022.391.9742)
Supplement: Translation

# Translation: Análisis de la distribución de macronutrientes en canastas alimentarias entregadas por las municipalidades durante la pandemia de COVID-19 en Perú

**Authors:** Bladimir Morales-Cahuancama, Gandy Dolores-Maldonado, Paul Hinojosa-Mamani, William Bautista-Olortegui, Cinthia Quispe-Gala, Lucio Huamán-Espino, Juan Pablo Aparco

**Affiliations:** 1 Centro Nacional de Alimentación y Nutrición, Instituto Nacional de Salud, Lima, Perú.; 2 Universidad Privada de Ciencias Aplicadas, Lima, Perú.; 3 Escuela Profesional de Nutrición, Facultad de Medicina, Universidad Nacional Mayor de San Marcos, Lima, Perú.; 4 Oficina General de Investigación y Transferencia Tecnológica, Instituto Nacional de Salud, Lima, Perú.

**Keywords:** Canasta alimentaria, Covid-19, Alimentación de Emergencia, Vulnerabilidad ante Desastre, Seguridad Alimentaria, Perú

## Abstract

**Objetivos.:**

Evaluar y comparar la distribución de macronutrientes de las canastas alimentarias entregadas por las municipalidades peruanas durante la pandemia de COVID-19 según dominio geográfico y nivel de presupuesto asignado.

**Materiales y métodos.:**

Análisis secundario de la base de datos **«**Consulta de adquisición y distribución de bienes de primera necesidad de la canasta básica familiar**»** de la Contraloría General de la República. Se realizó un muestreo probabilístico estratificado. Se calculó la distribución del aporte calórico según macronutrientes y se comparó con los intervalos aceptables de distribución de macronutrientes (IADM) del Instituto de Nutrición de Centro América y Panamá (INCAP) y del Instituto Nacional de Defensa Civil (INDECI).

**Resultados.:**

A nivel nacional la mediana del aporte calórico de las proteínas fue 7,7%; carbohidratos 62,5%, y grasas 28,1%. La proporción de municipalidades con canastas con déficit de proteínas fue de 84%. Las municipalidades con canastas con exceso de carbohidratos variaron de 16,5% (según INCAP) a 35,9% (según INDECI) y con exceso de grasas fue 61,6 % (según INCAP) y 20,2% (según INDECI). Según INDECI, a nivel nacional solo el 9,2% de municipalidades entregaron canastas con distribución adecuada de macronutrientes, aquí destaca Lima Metropolitana con las proporciones más altas; mientras que en la selva este porcentaje fue menor del 5%.

**Conclusiones.:**

La mayoría de las canastas entregadas no tuvieron distribución adecuada de macronutrientes, en especial las canastas de municipalidades fuera de Lima Metropolitana o las que contaban con menor presupuesto. Los carbohidratos y las grasas fueron los nutrientes que se incluyeron en exceso, mientras que las proteínas tuvieron déficit.

## INTRODUCCIÓN

La pandemia por la COVID-19 ha afectado considerablemente los sistemas sanitarios y la calidad de vida de la población mundial ^1^. El 16 de marzo de 2020 el Gobierno Peruano declaró el estado de emergencia a nivel nacional e impuso el aislamiento social ^2^ lo cual limitó las actividades económicas y agudizó la vulnerabilidad social de muchos hogares ^3^. Además, la pandemia afectó el acceso a los alimentos y los mercados se convirtieron en focos de infección, por lo que se dispuso el cierre o limitación del aforo. Asimismo, la disminución de ingresos en las familias y la especulación de precios afectaron el acceso a los alimentos ^4^. Estos cambios agravaron la inseguridad alimentaria (IA) en el Perú, que antes de la pandemia ya era un problema complejo ^5^; así, para el período 2014-2016 la prevalencia de IA se estimó en 50,7% y para 2018-2020 aumentó a 67% ^6^, aunque otros autores han estimado hasta 83% de IA en el 2020 ^7^.

Frente a este panorama de crisis económica e IA por la pandemia, el gobierno central transfirió 213 millones de soles, equivalente a 59,6 millones USD (tipo de cambio a julio de 2020) a las municipalidades para la adquisición de alimentos de la canasta básica familiar, con la finalidad de atender, por única vez, a los hogares en situación de vulnerabilidad ^8^. La distribución del dinero fue proporcional a la cantidad de habitantes de cada municipalidad. Asimismo, se publicaron los Lineamientos para la Organización y Distribución de las Canastas Alimentarias (LODCA) para orientar a las municipalidades sobre los detalles del programa ^9^ y actualmente recomienda la inclusión alimentos según la zona geográfica y los hábitos de consumo de la población.

La asistencia alimentaria como respuesta a crisis humanitaria ha demostrado ser beneficiosa para las poblaciones afectadas ^10^ ^,^ ^11^. Entidades como el Instituto de Nutrición de Centro América y Panamá (INCAP) ^12^ y el Instituto Nacional de Defensa Civil (INDECI) ^13^ recomiendan la composición de canastas de emergencia basadas en criterios alimentario-nutricionales, usando intervalos aceptables de distribución de macronutrientes (IADM) que expresan las recomendaciones de proteínas, carbohidratos y grasas, como porcentaje de la ingesta calórica total. Los IADM se estiman para garantizar una ingesta suficiente de nutrientes, sin aumentar los riesgos de enfermedades crónicas, y permite comprender si la asistencia alimentaria es nutricionalmente balanceada, propósito del análisis ^14^.

A pesar de que la pandemia por COVID-19 continúa y que es latente la necesidad de diversas intervenciones, entre ellas la distribución de canastas de alimentos de emergencia, a la fecha no se ha evaluado la composición nutricional, ni la adecuación de las canastas alimentarias entregadas frente a la emergencia sanitaria. Por tanto, la presente investigación tuvo como objetivo evaluar y comparar la distribución de macronutrientes de las canastas alimentarias entregadas por las municipalidades durante la pandemia de COVID-19 según dominio geográfico y nivel de presupuesto asignado.

**MENSAJES CLAVE**

Motivación para realizar el estudio: el criterio nutricional debe ser incorporado en la confección de las canastas alimentarias distribuidas a la población con limitado acceso alimentario.

Principales hallazgos: la mayoría de las canastas alimentarias de los municipios no estuvieron nutricionalmente balanceadas. Los carbohidratos y las grasas fueron los nutrientes que se incluyeron en exceso, mientras que las proteínas estuvieron en déficit.

Implicancias: es necesario mejorar las pautas para la confección de las canastas alimentarias de emergencia considerando los intervalos aceptables de distribución de macronutrientes y asegurar una canasta alimentaria adecuada; asimismo, buscar estrategias para incrementar la cantidad de alimentos fuentes de proteínas en las canastas de emergencias y regular la cantidad de grasas.

## MATERIALES Y MÉTODOS

### Diseño de estudio

El presente artículo es un análisis secundario de la base de datos «Adquisición y distribución de bienes de primera necesidad de la canasta básica familiar» de la Contraloría General de la República del Perú (CGRP) ^15^. 

### Población, muestra y muestreo

La población del estudio la conformaron las 1874 municipalidades registradas en la página web de la CGRP. El cálculo del tamaño de la muestra se hizo en base a una proporción esperada del 50% (máxima varianza), obteniéndose una muestra neta de 319 municipalidades; además, se adicionó un 30% por pérdida (municipalidades con errores en el registro de canastas).

Para la selección de las municipalidades se utilizó un muestreo probabilístico estratificado según ámbito geográfico: resto de costa, sierra, selva y Lima Metropolitana (diferenciado de costa). Además, se clasificaron según el monto de dinero asignado ^9^: Grupo A (50 000 soles o 14 000 USD); Grupo B (100 000 soles o 28 000 USD); Grupo C (200 000 soles o 56 000 USD) y Grupo D (mayor a 500 000 soles o 140 000 USD).

En cada municipalidad seleccionada se incluyó solo una canasta de alimentos que cumpliera con el criterio de inclusión: canastas de alimentos elaboradas con tres alimentos como mínimo; en el caso de que la municipalidad tuviera más de un tipo de canasta de alimentos se eligió la que se distribuyó a la mayor cantidad de beneficiarios. Se excluyeron las canastas con registros con errores, como aquellas que incluyeron alimentos en cantidades excesivas, información con valores extremos de pesos o volúmenes de alimentos. El 53% de municipalidades seleccionadas tuvo más de un tipo de canasta (Figura 1).

**Figura 1:** Flujograma de recolección de información de las canastas alimentarias de los municipios seleccionados.

### Procedimientos

La extracción de la información de la web (desde el 17 al 21 de junio de 2020) se realizó después del plazo límite que tuvieron las municipalidades para registrar la información (30 de mayo de 2020). Posteriormente, cuatro nutricionistas hicieron el control de calidad de la información recopilada para verificar los pesos de los alimentos y la información completa. Con la información disponible se acondicionó una base de datos que registraba el nombre, y el peso o volumen del alimento.

En el caso de alimentos que se registraron en la web como «unidades» (sin informar el peso) se revisó el precio de cada unidad y se aproximó el peso considerando el costo promedio del producto en mercados mayoristas y minoristas ^16^. Se encontró que la mayoría de las aproximaciones de peso se concentraron en las carnes (52,2%), debido a que varias conservas de pescado no fueron registradas con el peso neto del producto sino en «unidades». Luego se asignaron códigos a cada alimento según las Tablas Peruanas de Composición de Alimentos (TPCA) ^17^; algunos alimentos no tenían código en la TPCA, y se les asignó el código de un alimento similar (aproximación por similitud). La mayoría de las aproximaciones por similitud también se concentraron en las carnes (67,5%), puesto que muchas variedades de conservas de pescado no tienen un código específico en la TPCA.

Luego de obtener los códigos TPCA y cantidades (gramos o litros) de todos los alimentos, se calculó la cantidad de energía (kcal), proteínas (g), grasas (g) y carbohidratos (g) que contenía cada alimento. Posteriormente, se calculó la cantidad de energía y los tres macronutrientes (g) de cada canasta sumando los aportes de todos los alimentos sin excluir ninguno.

Asimismo, se clasificó los alimentos según grupos recomendados por la «Guía alimentarias para la Población Peruana» ^18^: azúcares, carnes, cereales, aceites, lácteos, leguminosas, frutas, huevo, verduras y tubérculos.

### Variables y mediciones

La distribución del aporte calórico según macronutrientes se calculó como el cociente del aporte energético de los macronutrientes de la canasta, entre el total de calorías de la canasta, multiplicado por 100 para obtener el porcentaje; además, para estimar la energía disponible se aplicaron los factores de Atwater para proteínas, grasas y carbohidratos: 4, 9 y 4 kcal respectivamente ^19^. Para evaluar si las canastas tuvieron una composición alimentaria balanceada, se comparó la distribución del aporte calórico de macronutrientes según los IADM internacionales del INCAP que recomienda un contenido de proteínas 10-15% del contenido energético total (kilocalorías), grasas 20-25%, y carbohidratos 60-70% ^12^; y los IADM nacionales de INDECI que sugiere el aporte de proteínas de 10-15% del contenido energético total, grasas 20-35%, y carbohidratos 50-65% ^13^.

### Análisis estadístico

Se calcularon las frecuencias absolutas y relativas para las variables categóricas, en tanto que en las cuantitativas los resultados se expresaron como mediana y rango intercuartílico. Además, se aplicó la prueba de Kolmogórov-Smirnov a las variables: «aporte calórico de macronutrientes» y «cantidad de grupos de alimentos», las cuales no presentaron una distribución normal. Para comparar el aporte calórico y la cantidad de grupos de alimentos según la ubicación geográfica y tipo de presupuesto asignado se utilizó la Prueba de Kruskal-Wallis, con comparaciones entre grupos *post hoc* mediante la prueba de Dunnett. Se tomó como significación estadística p<0,05. Para el análisis estadístico se utilizó el programa informático SPSS 25.0.1.

### Aspectos éticos

El presente estudio corresponde a un estudio de base de datos secundaria pública. Se accedió a la información del «Reporte oficial de compra y entrega canasta básica familiar» de la página web de la CGRP disponible en https://emergenciasanitaria.contraloria.gob.pe/ ^15^.

## RESULTADOS

El estudio inició con 415 municipalidades y al aplicar los criterios de exclusión se obtuvieron 366 municipalidades con su respectiva canasta alimentaria, lo que permitió superar el tamaño neto de la muestra (Figura 1).

Las proteínas tuvieron una mediana de aporte calórico de 7,7%, los carbohidratos 62,5% y las grasas 28,1% del total de calorías. Según dominio geográfico, la sierra y la selva tuvieron las canastas con menor aporte calórico de proteínas con diferencias significativas al compararse ambas con Lima Metropolitana y resto de costa (p<0,001). Lima Metropolitana mostró significativamente el menor aporte calórico de carbohidratos (p<0,05). En grasas, el resto de costa tuvo el menor aporte calórico en comparación al resto del Perú (p<0,05). Por otra parte, las municipalidades con mayor presupuesto (Grupo D) tuvieron significativamente mayor aporte calórico de proteínas y menor aporte de carbohidratos (p<0,05); mientras que las municipalidades del Grupo B mostraron significativamente el mayor aporte calórico proveniente de las grasas (Tabla 1).

**Tabla 1:** Porcentaje del aporte calórico de las canastas alimentarias distribuidas por municipalidades según dominio geográfico y cantidad de presupuesto.

Municipalidades	Aporte calórico por macronutrientes		
	Proteínas	Carbohidratos	Grasas
	Me (RIC)	Me (RIC)	Me (RIC)
Dominio geográfico			
Resto costa	8,5 (7,4 - 9,6) ^a^	62,3 (58,6 - 67,0) ^a^	26,3 (21,2 - 31,4) ^a^
Sierra	7,4 (5,8 - 8,8) ^b^	62,9 (56,9 - 67,9) ^a^	28,5 (21,4 - 33,8) ^b^
Selva	7,4 (6,0 - 8,8) ^b^	62,3 (57,8 - 67,7) ^a^	28,1 (23,1 - 34,0) ^b^
Lima Metropolitana	10,7 (9,3 - 11,3) ^c^	57,8 (54,4 - 61,5) ^b^	29,4 (24,7 - 30,6) ^ab^
Presupuesto			
Grupo A (14 000 USD)	7,7 (6,1 - 8,9) ^a^	63,8 (57,8 - 68,4) ^a^	27,3 (21,5 - 33,2) ^a^
Grupo B (28 000 USD)	7,4 (5,9 - 9,0) ^a^	61,6 (55,1 - 67,1) ^b^	30,1 (22,2 - 35,2) ^b^
Grupo C (56 000 USD)	7,6 (6,4 - 9,5) ^a^	62,8 (57,9 - 66,8) ^ab^	27,3 (23,3 - 31,6) ^a^
Grupo D (>140 000 USD)	10,7 (9,0 - 11,6) ^b^	58,8 (54,1 - 61,5) ^c^	25,0 (24,2 - 29,4) ^a^
Nacional	7,7 (6,2 - 9,2)	62,5 (57,1 - 67,4)	28,1 (21,8 - 33,7)

Kruskal-Wallis, prueba de *post-hoc *de Dunnett. Los valores con letras diferentes representan diferencias significativas entre los grupos de comparación, p<0,05

Me: mediana, RIC: rango intercuartílico

En la Tabla 2, se analizaron las cantidades según grupos de alimentos. En todas las canastas las carnes fueron los alimentos en menor cantidad (0,7 kg); mientras que los cereales estuvieron en mayor cantidad (13,1 kg). Las municipalidades de Lima Metropolitana brindaron la menor cantidad de azúcar en sus canastas, en tanto que en la sierra incorporaron mayor cantidad de azúcar, 1,5 kg (p<0,05). El resto de costa y la selva incluyeron las menores cantidades de carne comparadas con los otros dominios, 0,7 kg (p<0,05). En cuanto al aceite, la sierra y la selva lo incluyeron en mayor cantidad (p<0,05). La sierra incluyó la mayor cantidad de leguminosas en sus canastas, 3 kg. Las canastas de las municipalidades con menor presupuesto (Grupo A) tuvieron mayor cantidad de azúcar, cereales, aceites, lácteos y leguminosas, con diferencias significativas con las municipalidades de mayor presupuesto (p<0,05).

**Tabla 2:** Cantidad por grupo de alimentos de las canastas alimentarias según dominio geográfico y cantidad de presupuesto

Municipalidades	Azúcar (kg)	Carnes* (kg)	Cereal (kg)	Aceites (L)	Lácteos** (kg)	Leguminosas (kg)
	Me (RIC)	Me (RIC)	Me (RIC)	Me (RIC)	Me (RIC)	Me (RIC)
Dominio geográfico						
Resto de Costa	3,0 (2,0 - 5,0) ^a^	0,7 (0,5 - 0,9) ^a^	11,5 (9,3 - 13,4) ^a^	1,0 (1,0 - 2,0) ^a^	1,6 (1,2 - 2,0) ^a^	2,0 (1,0 - 2,5) ^a^
Sierra	5,0 (3,8 - 7,0) ^b^	0,8 (0,5 - 1,2) ^b^	14,3 (11,0 - 19,0) ^b^	2,0 (1,5 - 3,0) ^b^	1,6 (1,2 - 2,4) ^a^	3,0 (2,0 - 4,0) ^b^
Selva	3,0 (3,0 - 5,0) ^a^	0,7 (0,5 - 0,9) ^a^	13,0 (10,9 - 16,0) ^c^	2,0 (1,0 - 2,0) ^c^	1,2 (1,0 - 2,0) ^ab^	2,0 (1,0 - 3,0) ^a^
Lima Metropolitana	1,5 (1,0 - 2,0) ^c^	1,0 (1,0 - 1,2) ^c^	10,0 (7,5 - 10,4) ^d^	1,0 (1,0 - 2,0) ^a^	1,6 (1,2 - 2,0) ^a^	2,0 (2,0 - 2,3) ^a^
Presupuesto						
Grupo A (14 000 USD)	5,0 (4,0 - 8,0) ^a^	0,8 (0,5 - 1,2) ^a^	14,5 (11,0 - 20,0) ^a^	2,0 (1,5 - 3,0) ^a^	1,6 (1,2 - 2,4) ^a^	2,6 (2,0 - 4,0) ^a^
Grupo B (28 000 USD)	4,0 (3,0 - 5,7) ^b^	0,8 (0,5 - 1,0) ^ab^	13,4 (11,0 - 16,0) ^b^	2,0 (1,0 - 3,0) ^a^	1,6 (1,2 - 2,0) ^a^	2,0 (2,0 - 3,3) ^a^
Grupo C (56 000 USD)	3,0 (2,0 - 5,0) ^c^	0,7 (0,5 - 1,0) ^c^	12,0 (9,0 - 14,0) ^c^	1,8 (1,0 - 2,0) ^b^	1,2 (0,8 - 1,6) ^b^	2,0 (1,8 - 3,0) ^b^
Grupo D (>140 000 USD)	1,5 (1,0 - 2,0) ^d^	0,9 (0,7 - 1,0) ^a^	9,5 (8,0 - 10,0) ^d^	1,0 (1,0 - 1,0) ^c^	1,6 (1,2 - 2,0) ^ab^	2,0 (2,0 - 4,0) ^ab^
Nacional	5,0 (3,0 - 6,0)	0,7 (0,5 - 1,0)	13,1 (10,0 - 17,0)	2,0 (1,0 - 3,0)	1,6 (1,2 - 2,0)	2,0 (2,0 - 4,0)

*Kruskal-Wallis*, prueba de* post-hoc *Dunnet. Los valores con letras diferentes representan diferencias significativas entre los grupos de comparación, p<0,05

Me: mediana. RIC: rango intercuartílico.

*El grupo carnes estuvo conformado en su mayoría por conservas de pescado enlatadas.

**El grupo de lácteos estuvo conformado en su mayoría por leche evaporada en tarro. Solo para efectos de la presentación, no se incluyeron en la Tabla 2 las columnas referentes a los grupos de alimentos: frutas, huevo, verduras y tubérculos porque muy pocas canastas incluyeron estos alimentos y en muchas categorías las medianas fueron “0”. Todos los alimentos fueron incluidos en el cálculo de aporte calórico y de macronutrientes.

Según los IADM de INCAP e INDECI la proporción de municipalidades con déficit de proteínas fue de 84%, mientras que el déficit de carbohidratos y grasas fue de 39,8 y 18,1% según INCAP y de 7,5 y 18,1 % según INDECI. No se encontró exceso en proteínas, pero en carbohidratos el exceso varió de 16,5 a 35,9%, según criterio aplicado. De igual forma en grasas, el exceso estuvo entre 20,2 y 61,6 % (Tabla 3).

**Tabla 3:** Adecuación a estándares INCAP e INDECI del aporte calórico de macronutrientes según dominio geográfico y cantidad de presupuesto

Municipalidades	INCAP / INDECI	INCAP		INDECI		INCAP		INDECI	
	Déficit en proteínas n (%)	Déficit de carbohidratos n (%)	Exceso de carbohidratos n (%)	Déficit de carbohidratos n (%)	Exceso de carbohidratos n (%)	Déficit de grasas n (%)	Exceso de grasas n (%)	Déficit de grasas n (%)	Exceso de grasas n (%)
Dominio geográfico									
Resto de costa	228 (82)	93 (33,6)	39 (13,9)	19 (6,8)	95 (34)	57 (20,7)	155 (55,7)	57 (20,7)	43 (15,3)
Sierra	1067 (86)	498 (40,2)	247 (19,9)	113 (9,1)	471 (38)	248 (20)	780 (62,9)	248 (20)	254 (20,5)
Selva	266 (86,9)	121 (39,4)	24 (7,9)	6 (1,9)	102 (33,3)	35 (11,4)	189 (61,9)	35 (11,4)	73 (23,8)
Lima Metropolitana	15 (29,8)	35 (70,4)	0 (0)	4 (8,8)	6 (12)	0 (0)	31 (61,6)	0 (0)	9 (18,8)
Presupuesto									
Grupo A (14 000 USD)	790 (86,9)	330 (36,3)	179 (19,7)	63 (6,9)	375 (41,2)	180 (19,8)	547 (60,1)	180 (19,8)	176 (19,4)
Grupo B (28 000 USD)	419 (86,5)	219 (45,2)	71 (14,7)	58 (12)	156 (32,3)	96 (19,8)	320 (66,1)	96 (19,8)	127 (26,2)
Grupo C (56 000 USD)	350 (80,7)	167 (38,5)	55 (12,6)	16 (3,7)	137 (31,6)	60 (13,7)	265 (61,1)	60 (13,7)	65 (15)
Grupo D (>140 000 USD)	17 (36)	32 (67,4)	6 (11,7)	4 (9,4)	6 (11,7)	6 (11,7)	23 (48,5)	6 (11,7)	11 (24,3)
Nacional	1576 (84)	748 (39,8)	310 (16,5)	142 (7,5)	674 (35,9)	340 (18,1)	1155 (61,6)	340 (18,1)	379 (20,2)

Resumen de las IADMI según INCAP e INDECI:

Déficit de proteínas INCAP e INDECI (<10%)

Déficit de carbohidratos INCAP (<60%); Exceso de carbohidratos INCAP (>70%)

Déficit de carbohidratos INDECI (<50%); Exceso de carbohidratos INDECI (>65%)

Déficit de grasas INCAP (<20%); Exceso de grasas INCAP (>25%)

Déficit de grasas INDECI (<20%); Exceso de grasas INDECI (>35%)

Respecto a la ubicación geográfica, en Lima Metropolitana el déficit de proteínas fue del 29,8%, mientras que en las otras zonas fue mayor de 80%. En cuanto a carbohidratos, Lima Metropolitana tuvo 70,4% de déficit y ninguna canasta presentó exceso de carbohidratos (según INCAP). Respecto a grasas, solo las municipalidades de Lima Metropolitana no tuvieron déficit, mientras que el aporte excesivo de grasas se observó en todos los dominios geográficos. También se aprecia que las municipalidades con mayor asignación presupuestal (Grupo D) tuvieron menos problemas de déficit de proteínas, exceso de carbohidratos y exceso de grasas, en comparación con las municipalidades con menos presupuesto (Tabla 3).

En la Figura 2 se observa que, según los criterios de INDECI, a nivel nacional solo el 9,2% de municipalidades entregaron canastas con distribución adecuada de los tres macronutrientes; destacando Lima Metropolitana con las proporciones más altas de canastas adecuadas, mientras en la selva este porcentaje fue menor del 5%. Al aplicar el parámetro de INCAP menos del 5% del total de canastas cumplían con los IADM, mientras que el resto de costa fue el dominio con menor adecuación con solo 3,3% de canastas adecuadas.

**Figura 2:** Porcentaje de canastas alimentarias con adecuada distribución de carbohidratos, proteínas y grasas, según dominio geográfico.

La Figura 3 muestra el porcentaje de canastas que cumplieron con la adecuada distribución de los tres macronutrientes según nivel de presupuesto, y se observa que las municipalidades con menor presupuesto tuvieron las proporciones más bajas de adecuación. Según los criterios de INDECI el 64% de municipalidades del Grupo D distribuyeron canastas adecuadas, mientras que en el grupo A y B solo el 6,5 y 5% cumplieron los IADM; al aplicar los parámetros de INCAP se observa que destacan los mismos grupos de municipalidades.

**Figura 3:** Porcentaje de canastas alimentarias con adecuada distribución de carbohidratos, proteínas y grasas según nivel de presupuesto.

## DISCUSIÓN

El estudio se realizó con el objetivo de evaluar la distribución de macronutrientes como energía, grasas y proteínas en las canastas alimentarias distribuidas por los gobiernos locales para atender la IA por la pandemia de COVID-19. Los hallazgos muestran que a nivel nacional menos del 10% de municipalidades evaluadas entregaron canastas que cumplían con los IADM, según INDECI; aunque al aplicar el criterio de INCAP el cumplimiento se redujo a menos del 5%. Según dominio geográfico las municipalidades de selva y sierra tuvieron la menor adecuación de macronutrientes; mientras que a nivel de presupuesto las municipalidades de los grupos A y B (con menor presupuesto) no superaban el 7% de canastas distribuidas con adecuación de macronutrientes.

Este sería el primer estudio que analiza el cumplimiento de los IADM en las canastas de alimentos entregados durante la pandemia del COVID-19 en el Perú. Algunos estudios, sobre canastas alimentarias realizados antes de la pandemia enfocaron su evaluación en proponer nuevas canastas básicas de alimentos (CBA); así, un estudio en Costa Rica en el 2013, comparó el perfil nutricional de los alimentos más consumidos (CBA real) con las recomendaciones de ingesta diaria de nutrientes para elaborar una propuesta optimizada (CBA recomendada) ^20^, de la misma forma, en Guatemala se construyó una nueva CBA para adecuar el consumo de nutrientes. Otros estudios, también antes de la pandemia, analizaron las CBA desde la perspectiva económica reportando la variación del precio de la CBA y sus causas ^21^ ^) ^o el costo de una CBA saludable ^22^

Los resultados del estudio muestran que la mayoría de las municipalidades no cumplieron con una adecuada distribución de macronutrientes y que existen diferencias entre las canastas distribuidas según ámbito geográfico y nivel de presupuesto. Respecto a la distribución inadecuada de macronutrientes, en las municipalidades fuera de Lima Metropolitana y con presupuestos menores destacaron las proteínas como el macronutriente más deficitario, con proporciones de déficit por encima del 80% (89 y 86%, respectivamente); estas mismas municipalidades mostraron el mayor porcentaje de exceso de carbohidratos. Por su parte, las canastas elaboradas por las municipalidades de Lima Metropolitana y las de mayor presupuesto se caracterizaron por un mayor aporte calórico de proteínas y menor aporte de calorías proveniente de carbohidratos.

En cuanto a cantidad de alimentos, las canastas de sierra y selva destacaron por tener mayor cantidad de azúcar, cereal y aceites. Similar comportamiento tuvo las municipalidades con menor presupuesto. Las municipalidades brindaron casi la misma cantidad de carnes, sin embargo, al incorporar alimentos como el azúcar y el aceite vegetal, alimentos sin aporte de otros nutrientes, aparte de calorías, alteraron el equilibrio de macronutrientes en desmedro de las proteínas. Esta situación hizo que las municipalidades que brindaron mayor cantidad de alimentos tuvieran menos adecuación de macronutrientes.

Además, es importante mencionar la diferencia en la adecuación de macronutrientes de las canastas según presupuesto asignado a las municipalidades, considerando que las municipalidades con mayor presupuesto tienen mayor personal responsable de la gestión. Los resultados podrían explicar una disminución en la calidad del gasto mientras más pequeña sea la municipalidad. Esta situación podría explicarse porque los equipos de gestión suelen ser limitados, así como la oferta de alimentos locales.

Los alimentos que aportan más proteínas como las carnes, leche y huevos; fueron los menos utilizados. Si bien los alimentos de origen animal tienen un costo elevado comparado con los vegetales, se podría mejorar la compra de estos alimentos optando por presentaciones más económicas, como las conservas en envases grandes, o leche en recipientes más baratos que la lata. Asimismo, es necesario desarrollar otras alternativas de alimentos de origen animal que puedan ser seguros para transportar, menor costo de envase o cambiar el tipo de carne a uno más económico.

Otro aspecto para destacar son las diferencias de los IADM entre los estándares que sirvieron en el análisis. Ambos IADM son similares para la recomendación de proteínas; sin embargo, INCAP tiene un rango más tolerable con los carbohidratos, e INDECI con las grasas. Si bien los IADM de INCAP fueron elaborados como un instrumento para la definición de la línea de pobreza, estos también son utilizados para estimar los requerimientos de la asistencia alimentaria en situaciones de emergencia. Y en su elaboración se tomó en cuenta sugerencias de personas responsables de la elaboración de CBA en distintos países. Mientras los IADM de INDECI fueron elaborados en función de la respuesta frente a emergencias o desastres a nivel nacional, con la colaboración de instituciones internacionales como el Programa Mundial de Alimentos (PMA).

Un aspecto importante para considerar es que el contexto actual de IA es diferente a escenarios previos de IA crónica por afectación de la accesibilidad a alimentos o IA transitoria por eventos políticos, sociales o naturales que interrumpen la disponibilidad y acceso a alimentos ^23^. La aparición repentina de la pandemia por COVID-19 impuso tres dinámicas interrelacionadas que afectaron la seguridad alimentaria y ocasionaron la crisis: interrupción de las cadenas de suministro de alimentos a todo nivel, la pérdida de ingresos en la población, sobre todo en los más pobres y el aumento de precios de los alimentos por las interrupciones, la especulación de comerciantes y compras excesivas por ansiedad ^24^. En este contexto, la distribución de canastas de alimentos sería más efectiva que la transferencia de dinero a través de bonos económicos, dado que la dinámica de la pandemia afectó no solo el acceso a alimentos sino también la disponibilidad, por tanto, tener más dinero no resolvería la escasez de alimentos ^25^.

Asimismo, es necesario considerar que las canastas alimentarias distribuidas en respuesta a la emergencia sanitaria por COVID-19 no tuvieron como finalidad cubrir los requerimientos nutricionales diarios de las familias beneficiarias, a diferencia de las canastas de emergencias propuestas por INCAP ^12^ o INDECI ^13^, que buscan cubrir los requerimientos en IA transitoria por desastres naturales y eventos climáticos en zonas focalizadas; estas circunstancias son diferentes al escenario de la pandemia por COVID-19 que afectó los sistemas alimentarios y redujo los ingresos económicos en todo el país, con efectos por largo tiempo. Sin embargo, los criterios de evaluación de distribución de macronutrientes empleados en el presente estudio son lineamientos disponibles de instituciones con experiencia internacional en la formulación de canastas alimentarias para emergencias.

En un contexto dónde los riesgos de complicaciones y mortalidad por COVID-19 están relacionados con enfermedades concernientes al sobrepeso y obesidad ^26^, resulta necesario vigilar los mensajes e intervenciones alimentarias que se entregan a la población para evitar el incremento de las ECNT ^27^; en ese sentido, la entrega de canastas con exceso de azúcar y aceite podría tener un efecto comunicacional pernicioso para la población, así como fomentar la preparación y consumo de alimentos que privilegien los sabores dulces y las preparaciones en frituras o con alto contenido de grasa.

Bajo esa perspectiva, es importante diseñar CBA adecuadas nutricionalmente para guiar el consumo de alimentos en la población, canastas de alimentos para emergencias por eventos climáticos o desastres naturales y otras modalidades de entrega de alimentos en condiciones de pandemia global que pueda provocar una nueva crisis alimentaria, así como la COVID-19, se han reportado probables escenarios similares de afectación de la seguridad alimentaria por virus de gripe aviar ^28^ y ébola ^29^.

Por otra parte, en la última cumbre para la asistencia alimentaria se hizo hincapié en la necesidad de innovar en el desarrollo de alimentos que mejoren aspectos críticos en la asistencia alimentaria como la baja ingesta de proteínas y el riesgo de enfermedades crónicas producto del consumo de alimentos altos en calorías ^30^. En el contexto nacional, se sugiere promover la innovación de productos alimenticios que ayuden a mejorar el perfil nutricional de las canastas. Al respecto, el balance de macronutrientes podría mejorarse con leguminosas (menestras) ya que son una buena fuente de proteína vegetal. Llama la atención su pobre inclusión en las canastas entregadas por las municipalidades, puesto que el mercado nacional está permanentemente abastecido debido a su amplia producción en diferentes zonas del país, especialmente en la costa norte y central del Perú ^31^. Además, esta situación podría mejorar dando soporte informativo para mejorar la confección de sus canastas. En los «Lineamientos para la Organización y Distribución de las Canastas Alimentarias», la información relacionada a la composición nutricional de la canasta fue muy limitada ^9^.

El estudio secundario de la base de datos conlleva a limitaciones debido a que los análisis estadísticos están supeditados a las variables disponibles y la calidad de la información; en nuestro caso, la información registrada sobre las canastas no tuvo un proceso de validación. Además, al momento de recoger la información de la página web de la CGRP, hubo algunas municipalidades que no registraron la información de sus canastas. Sin embargo, esto no afectó la representatividad del muestreo, que contempló una tasa de pérdidas, cumpliendo con el mínimo requerido. Asimismo, varias municipalidades registraron los alimentos de las canastas de forma imprecisa, por ese motivo fueron nutricionistas quienes determinaron el peso aproximado según el precio unitario del alimento y, en algunos casos, se descartó el análisis de la canasta cuando se encontraron muchos errores. Una limitación en el análisis de macronutrientes fue que no se pudo analizar el tipo de grasas y carbohidratos porque la TPCA no incluye esta información y las etiquetas nutricionales de los alimentos no brindan estos datos. Otra limitación fue que la evaluación de las canastas alimentarias no es un indicador directo del consumo de nutrientes entre los miembros de las familias. Debido a que la canasta no está destinada a cubrir los requerimientos nutricionales de cada individuo, estos pudieron adicionar otros alimentos a su dieta fuera de la canasta; además, no se verificó que las familias consumieran toda la canasta. Sin embargo, esta fue la única información sobre la respuesta alimentaria ante la emergencia por COVID-19 y permite aproximarnos a la situación nutricional.

En conclusión, el estudio señala que la mayoría de las canastas de las Municipalidades que no eran de Lima Metropolitana o contaban con menor presupuesto no tuvieron una adecuada distribución de macronutrientes; los carbohidratos y las grasas fueron los nutrientes que se incluyeron en exceso, mientras que las proteínas tuvieron un déficit superior al 80%. Es necesario revisar y mejorar los «Lineamientos para la Organización y Distribución de las Canastas Alimentarias en el Perú» para incluir las pautas de elaboración de canastas de emergencia considerando los IADM y asegurar una canasta alimentaria adecuada; asimismo, buscar estrategias para incrementar la cantidad de alimentos fuentes proteínas en las canastas de emergencias y regular la cantidad de grasas.
